# Urinary tract infections caused by Pseudomonas aeruginos among children in southern Poland

**DOI:** 10.1186/2047-2994-4-S1-P262

**Published:** 2015-06-16

**Authors:** M Pobiega, J Maciąg, A Chmielarczyk, D Romaniszyn, M Pomorska-Wesolowska, G Ziolkowski, J Wojkowska-Mach, PB Heczko

**Affiliations:** 1 Chair of Microbiology, Jagiellonian University Medical School, Kraków, Poland; 2 Institute of Dentistry, Jagiellonian University Medical College, Kraków, Poland; 3 Higher School of Medicine in Sosnowiec, Poland, Sosnowiec, Poland

## Introduction

Urinary tract infections (UTIs) are an important cause of morbidity and mortality during the first 2 years of life. Knowledge of the antimicrobial resistance patterns may help clinicians choosing the empirical treatment.

## Objectives

The aim was to analyze the resistance and virulence of PAR causing UTIs among children in Southern Poland.

## Methods

PCR-screening for *las*B, *exo*S, *pil*A, *apr*A and *pil*B and antimicrobial-susceptibility were performed. MDR was non-susceptible to one antimicrobial in ≥3 antimicrobial classes. Extensively-drug resistant (XDR) was susceptible to ≤2 antimicrobial classes. PCR-screening for VIM, IMP and KPC was performed.

## Results

Median age (Q1;Q3) of the population (25 children) was 1.5 year (1;3). The most prevalent virulence gene was *exo*S (92.3%), modulating bacterial phagocytosis and invasion into cells. *Las*B gene (degrades of human competent molecules), was present among 80.8% of the isolates. *Apr*A (aeruginolysin that degrades biologically important proteins) was present in 61.5%. *Pil*B gene was not detected. Of the isolates, 92% were susceptible to gentamycin, tobramycin or cefepime, 85% to amikacin. A large number of isolates were resistant to meropenem (38.5%) or imipenem (19.2%). All were susceptible to colistin. Two isolates were XDR, 1 was MBL-positive. No KPC, IMP, VIM were found.

## Conclusion

Empirical selection of the antibiotics should be based on the knowledge of local antimicrobial susceptibilities of pathogens rather than on universal guidelines to maximize the benefit for patients and minimize the risk of developing drug-resistance. In this study, gentamycin and beta-lactams were shown to be the most appropriate for UTIs empiric therapy among children. Supported by 2012/05/N/NZ7/00786

## Disclosure of interest

None declared.

